# Novel application of synchrotron x-ray computed tomography for ***ex-vivo*** imaging of subcutaneously injected polymeric microsphere suspension formulations

**DOI:** 10.1007/s11095-020-02825-9

**Published:** 2020-05-14

**Authors:** Claire Patterson, Dean Murphy, Sarah Irvine, Leigh Connor, Zahra Rattray

**Affiliations:** 1 Early Pharmaceutical Development, Pharmaceutical Sciences, R&D, AstraZeneca, Macclesfield, UK; 2 Present Address: Seda Pharmaceutical Development Services, The Biohub, Alderley Park, Alderley Edge, Cheshire, UK; 3 Pharmaceutical Technology and Development, R&D, AstraZeneca, Macclesfield, UK; 4 Diamond Light Source, Harwell science and innovation campus, Oxfordshire, UK; 5 Present Address: Strathclyde Institute of Pharmacy and Biomedical Sciences, University of Strathclyde, Glasgow, UK

**Keywords:** X-ray computed tomography, subcutaneous injection, microsphere, depot, imaging, vehicle

## Abstract

**Purpose:**

Subcutaneously or intramuscularly administered biodegradable microsphere formulations have been successfully exploited in the management of chronic conditions for over two decades, yet mechanistic understanding of the impact of formulation attributes on *in vivo* absorption rate from such systems is still in its infancy.

**Methods:**

Suspension formulation physicochemical attributes may impact particulate deposition in subcutaneous (*s.c.*) tissue. Hence, the utility of synchrotron X-ray micro-computed tomography (μCT) for assessment of spatial distribution of suspension formulation components (PLG microspheres and vehicle) was evaluated in a porcine *s.c.* tissue model. Optical imaging of dyed vehicle and subsequent microscopic assessment of microsphere deposition was performed in parallel to compare the two approaches.

**Results:**

Our findings demonstrate that synchrotron μCT can be applied to the assessment of microsphere and vehicle distribution in *s.c.* tissue, and that microspheres can also be visualised in the absence of contrast agent using this approach. The technique was deemed superior to optical imaging of macrotomy for the characterisation of microsphere deposition owing to its non-invasive nature and relatively rapid data acquisition time.

**Conclusions:**

The method outlined in this study provides a proof of concept feasibility for μCT application to determining the vehicle and suspended PLG microspheres fate following *s.c.* injection. A potential application for our findings is understanding the impact of injection, device and formulation variables on initial and temporal depot geometry in pre-clinical or ex-vivo models that can inform product design.

**Figure:**
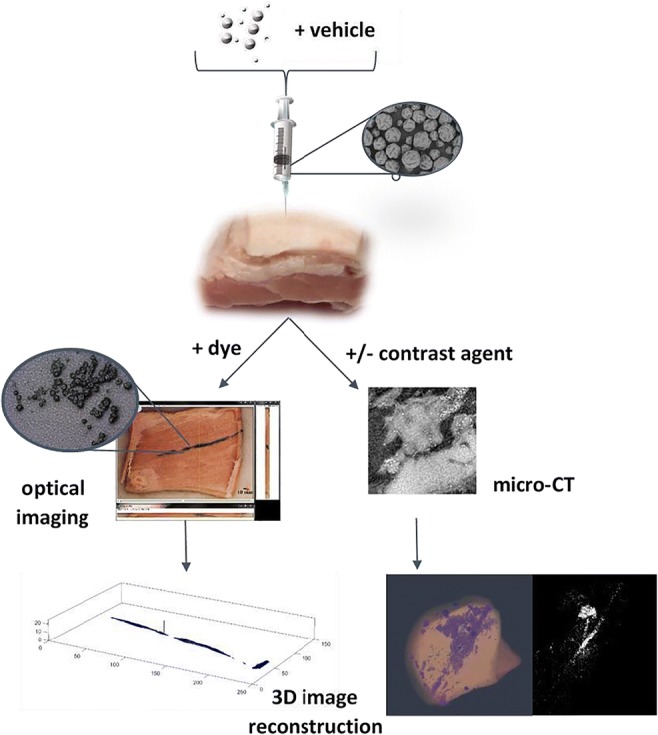
Graphical abstract

**Electronic supplementary material:**

The online version of this article (10.1007/s11095-020-02825-9) contains supplementary material, which is available to authorized users.

## Introduction

Self-administration of long acting formulations via subcutaneous *(s.c.)* or intramuscular (*i.m.*) injection is an attractive treatment paradigm in a range of chronic conditions (e.g. type 2 diabetes, schizophrenia, acromegaly), contributing to improved patient adherence and tolerability through reduction of peak-to-trough plasma concentration ratios. Encapsulation of active pharmaceutical ingredients (APIs) within biodegradable polylactide-co-glycolide (PLG) microspheres has been exploited commercially as a drug delivery platform providing sustained release of therapeutic agents following subcutaneous or intramuscular injection. Examples include BYDUREON® (AstraZeneca Pharmaceuticals) [1], RISPERDAL® CONSTA® (Janssen Pharmaceuticals) [2], VIVITROL® (Alkermes) [3], and Sandostatin LAR® (Novartis Pharmaceuticals) [4], which are all based on the Medisorb® microsphere technology (Alkermes, Inc.).

Factors governing the rate of appearance of API within the systemic circulation following subcutaneous injection of solutions have been extensively reviewed elsewhere [5–8]. Following injection, API is transported through the interstitium by diffusion and convection mechanisms before uptake by venous and/or lymphatic capillaries. The extent of spread and resultant interfacial surface area of the injected depot impacts i) the diffusional path length for the API ii) the interstitial pressure which influences convectional flow rate and iii) interaction with metabolic and immune processes within the subcutaneous tissue. Injection force, volume, viscosity, osmolality and interfacial tension have thus been cited as factors with the potential to affect rate (and extent) of API absorption [9].

Drug release from PLG microsphere suspensions is primarily governed by hydrolytic degradation of the microspheres [9]. Hence, in addition to understanding geometry of vehicle deposition, the spatial distribution of microspheres relative to suspending vehicle could be of direct relevance to microsphere *in vivo* performance. Additionally, study of the temporal fate of injected microspheres may shed light on the nature of particle deposition (single versus agglomerated mass) at the injection site, and anatomical localisation relative to the suspending vehicle (co-localisation versus sieving by the extracellular matrix), all of which contribute to mechanistic understanding of the impact of formulation, injection or physiological parameters on the rate of API absorption. A direct link between depot geometry of microsphere suspensions and pharmacokinetic outcomes, however, is yet to be established, attributable in part to the paucity of information on the former.

Label-free imaging techniques are generally preferred to circumvent inadvertent perturbation of formulation physicochemical properties. However, label-free techniques generally suffer from disadvantages such as the requirement for tissue excision and cryo-microtomy (e.g. mass spectrometry imaging), insufficient focal depth for *s.c.* injection sites (e.g. high frequency ultrasound, optical coherence tomography (OCT) [10, 11]), or lack of resolution for the identification of weakly attenuating small objects (such as PLG microspheres) (magnetic resonance imaging, MRI [12, 13], ultrasound) [14].

Herein, we describe a novel approach for simultaneously visualising the post-injection deposition of PLG microspheres and suspending vehicle in subcutaneous tissue using synchrotron X-ray micro-computed tomography (μCT). Data was collected both in the presence and absence of X-ray contrast agents. *Ex vivo* porcine abdominal tissue (≤12 h post mortem) was used, given its anatomical similarity to human subcutaneous tissue, and since tissue viability was less important for demonstration of technique feasibility. Data obtained from synchrotron μCT measurements are compared to a modified macrotomy technique adapted from Jockel et al. [15], and the relative merits of the two approaches discussed. Potential applications of this technique in the mechanistic understanding of API absorption from *s.c.* suspension formulations are presented.

## Materials and methods

### Materials

Porcine abdominal tissue was obtained (≤12 h post mortem) from a local abattoir from adult pigs that were not specifically slaughtered for this study.

PLG microspheres were prepared *in-house* using a coacervation method. Two suspending vehicles were selected with differing physicochemical attributes; a low viscosity (25 mPa.s at 20°C) non-aqueous vehicle, medium chain triglycerides (MCT), and a more viscous (122 mPa.s at 20°C) aqueous solution comprising 3% *w/v* carboxymethylcellulose sodium (CMC-Na) in phosphate-buffered saline containing 0.1% *w/v* polysorbate 20. MCT was obtained from Croda International (Goole, UK). CMC-Na, and colorimetric dyes (Patent Blue V and Solvent Green 3) were obtained from Sigma Aldrich (Gillingham, UK).

### Methods

#### Formulation preparation

For the μCT experiments, injections were performed in the presence and absence of X-ray contrast agent. Where indicated, a 10% *v/v* concentration of appropriate iodine-based contrast agent (Lugol’s solution for the aqueous vehicle and Lipiodol for the non-aqueous vehicle) was spiked into each formulation vehicle. Formulations for macrotomy and optical microscopy were spiked with 10% *v/v* (desired final volume) of colorimetric dye (Patent Blue V and Solvent Green 3 for the aqueous and nonaqueous vehicles, respectively).

Dyes and contrast agents were selected based on their solubility in the vehicle, hence the requirement for different dyes/contrast agents for the aqueous and non-aqueous formulations. A Mars Haake rheometer equipped with a C35/1° CSL (222–1885) cone and plate was used to confirm no impact of contrast agent on vehicle rheology at this concentration.

#### Microsphere characterisation Scanning Electron Microscopy (SEM)

The size and morphology of microspheres was assessed using scanning electron microscopy (SEM) prior to suspension in the formulation vehicle. The microspheres were sputter coated with <5 nm of gold using a Quorum Q150R S (Quorum Technologies UK Limited) and then SEM micrographs were acquired using a Hitachi TM-1000 SEM microscope at an accelerating voltage of 15 kV at magnifications of 500x and 5000x.

#### Particle size characterisation (Laser diffraction)

The particle size distribution of the microspheres was determined by laser light scattering employing a Beckman-Coulter LS 13320 Laser Diffraction Particle Size Analyzer consisting of a wet dispersion module, a laser, a focusing lens, a photosensitive detector, and a data processing unit.

#### Sample preparation

Abattoir-fresh porcine abdominal tissue was excised as either small (3 × 3 cm) or large (10 × 10 cm) sections that were subsequently injected with either contrast agent (Lugol’s solution or Lipiodol) or colorimetric dye (Solvent Green 3 or Patent Blue V) spiked formulation for μCT and macrotomy studies, respectively. Porcine abdominal tissue is widely considered the most relevant in terms of structure and composition to human *s.c.* tissue [16] and was therefore considered appropriate for use in the present study.

Injections were performed perpendicular to the tissue section, and the device (23G, 6 mm hypodermic needle) maintained *in situ* for 8–10 s to reduce backflow.

Tissue samples for μCT were embedded at ambient temperature in agarose gel (to minimise drying and associated shrinkage effects during scans) and imaged immediately following agarose setting (i.e. 20 min).

**Fig. 1:**
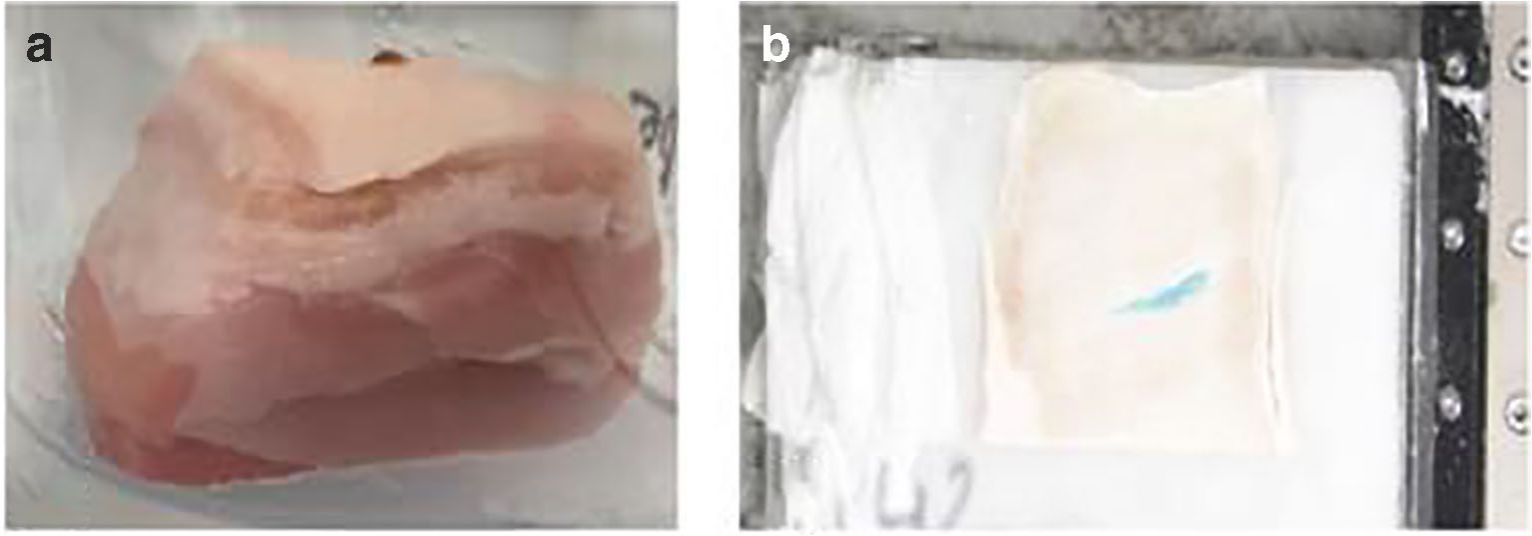
Porcine abdominal tissue injected with microsphere suspension and embedded in (A) agarose and (B) injected frozen tissue section undergoing macrotomy.

For macrotomy, injected tissue sections were flash-frozen and embedded in 2% *w/v* CMC paste to facilitate sectioning (Fig. 1). This was followed by reference point was inserted in each sample block to aid region of interest (ROI) alignment during image analysis. Following mounting in CMC paste and setting (i.e. 20–30 min), samples were stored until further analysis in a − 80°C freezer.

#### Synchrotron x-ray micro-computed tomography (μCT) image acquisition

X-ray synchrotron measurements were performed using beamline I12 at Diamond Light Source (Oxfordshire, UK) [17]. The beamline has built two detector systems with custom designed optics coupled to the commercially available PCO edge sCMOS camera. One detector system uses a single camera and a choice of four magnification lens to image the optical light created by the scintillator. For this experiment the largest field of view option was used (Module 1) and the magnification has an effective pixel size of 18.5 μm and a field of view (FOV) of 47 × 12 mm. The second detector (Large Field of View detector) uses two PCO edge cameras mounted side by side creating a single continuous field of view from two images stitched together. This arrangement has an effective pixel size of 21.5 μm and a field of view of 97 × 25 mm. Further details of the two modules are provided in the supplementary information section.

An incident X-ray beam energy of 70 keV was chosen to maximise flux and minimise absorption. The sample-to-detector distance was set to 1 m to generate propagation-based phase contrast (otherwise known as ‘edge-enhancement’) in the images [18]. For the collection of each tomographic dataset, 3600 projection images were acquired over an angular range of 180°. Exposure times were 1.25 ms for module 1 giving a scan time of 50 s and 40 ms for the LFV detector giving a scan time of 150 s. Each tissue sample was imaged 2–4 times in the vertical direction to create a stack of tomograms covering its full height. A short scan time was used to ensure minimal diffusion of vehicle during image acquisition.

#### Image pre-processing and reconstruction

All tomographic pre-processing and reconstruction steps were performed using in-house software and computing clusters at Diamond Light Source. The python-based modular reconstruction pipeline Savu [19] was used for the majority (a process flow diagram outlines the steps in Fig. S1 of the supplementary information section).

After the initial flat field correction, the LFV module projection images required stitching together, and the module 1 projection images were corrected for a slight lens distortion effect [20].

Following stitching/distortion correction, a raven ring removal filter was applied. GPU processed tomographic reconstruction based on the filtered back projection algorithm was then performed twice, separately for each data set: firstly, applying standard attenuation-based reconstruction and then as a second alternate phase-based reconstruction following an additional filtering step. That step computes filtered projections using the single image phase retrieval approach developed by Paganin et al. [21] and yields additional area contrast from the edge enhancement which is present in the unfiltered data.

Finally, for both process branches, reconstructed sections from the multiple scans corresponding to each sample were combined and renumbered to produce a single set of consecutively numbered sections for each sample.

#### Microsphere detection

Software for the semi-automated detection of PLG microspheres throughout the reconstructed tomographic section stacks were custom-written for this application within IDL 9.4 (Harris Geospatial Solutions). Following the initial manual inspection of the sections within each data set (using ImageJ) for the presence of microspheres, a cursor-interactive code was used to mark regions of interest (ROIs) within the stack of sections. These ROIs were passed as coordinates into an automated combined thresholding script which utilised both the standard and phase retrieved reconstructed data and applied a combination of intensity and texture thresholding (via a high pass filter) to define a binary map marking the central coordinates of each microsphere. Manual inspection was still required to check for false positives and to determine the thresholding values for each different sample. A schematic of the workflow is given in the supplementary materials section (Fig. S3).

#### Visualisation

Three-dimensional volume representations of the reconstructed data sets and marked microsphere distributions were generated within the three-dimensional visualisation and measurement software package, Avizo 9.4 (Thermo Fisher Scientific). Renderings of the contrast agent were generated after segmentation via simple intensity thresholding of the phase retrieved reconstructed data.

For the full manual segmentation of one representative small tissue sample, the full phase retrieved reconstructed dataset was used for the input, then downsized with 2 × 2 × 2 binning. Inside the segmentation editor of Avizo each of the main observed constituent materials (adipose and muscle layers, contrast agent and air bubbles) were labelled with a combination of thresholding and magic wand tools. Manual tracing of the very low contrast fat-agarose interface was aided with the use of a Wacom graphics tablet and then selection interpolation. The corresponding unbinned binary map of the sample microsphere distribution was added and convolved with a gaussian shape function before separate volume rendering. Movies were created through use of the Avizo animation editor.

#### Macrotomy

##### Macrotomy, digital photography and optical microscopy

In parallel to μCT measurements, a macrotomy method adapted from Jockel et al [22] was used to characterise vehicle and microsphere deposition for the same formulations. To aid vehicle detection, the aqueous and non-aqueous vehicles were spiked with Patent Blue V and Solvent Green 3, respectively.

Immediately post-injection, tissue samples were flash-frozen and embedded in 2% *w/v* CMC paste. Cryo-macrotomy was performed on the frozen tissue samples, and excised tissue sections of 30 μm thickness were affixed to a custom tape to aid visualisation with optical microscopy). For macroscopic characterisation of vehicle depot, images were captured using a Canon EOS digital camera fixed at the same height relative to the tissue block. Microscopic assessment of microsphere deposition in collected macrotomy sections was also performed using a Zeiss scope A1 optical microscope equipped with a 10x/0.25NA N-Achroplan objective lens. Multiple fields of view were acquired to assess the localisation of microspheres within tissue cross sections.

Optical images of tissue sections from samples injected with dyed formulation were processed in ImageJ (FIJI, v1.49p). All images were imported into ImageJ as a virtual stack and aligned relative to reference points. Subsequently, the image stack was converted (pixel size 0.353 mm) to three-dimensional stacks. Additionally, the voxel depth was calibrated as 180 μm to represent the frequency of sampling across the sample depth during sectioning. Subsequently, all images within the stack were thresholded manually to exclude the contribution of undyed regions (background tissue contribution to signal) to the measured area. The thresholded image stack was converted to a binary image to ease visualisation of regions stained by the vehicle depot. To validate co-localisation of the binary mask with dyed depot, image calculator was used. Subsequently, three-dimensional reconstructions of the binary stacks were rendered using an *in-house* MatLab package. Further processing e.g. quantification of % area stained by vehicle and volume of staining is possible using Image J.

## Results

### Microsphere characterisation

**Fig. 2:**
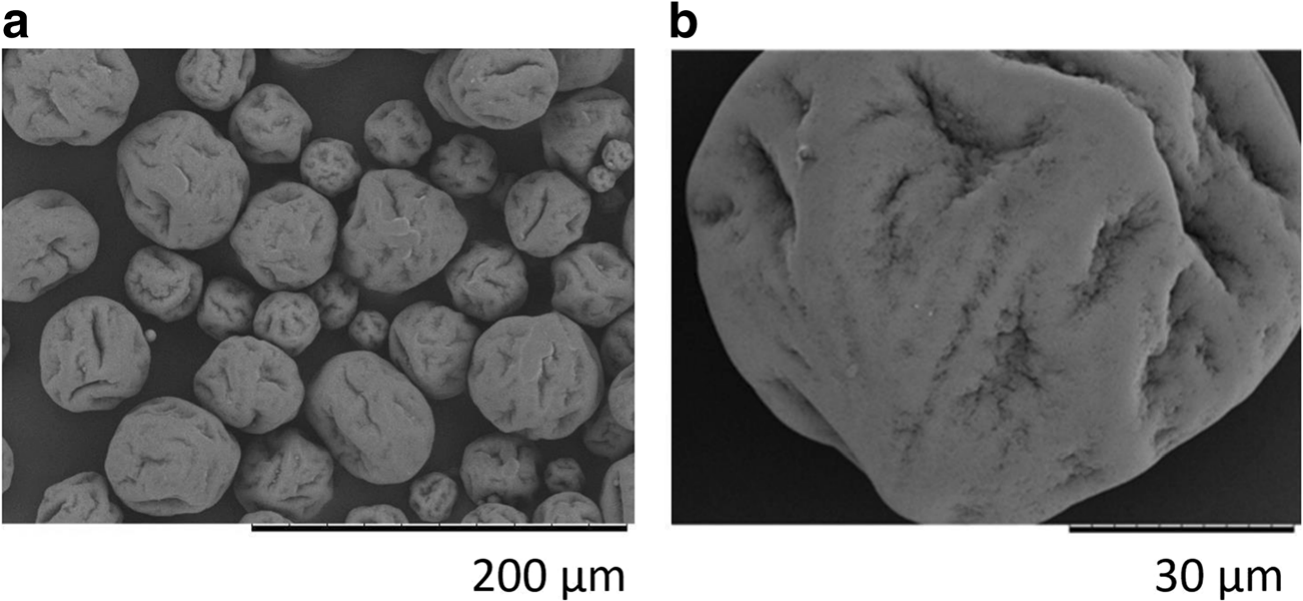
Representative SEM micrographs of dry PLG microsphere morphology. (A) 500x magnification (B) 2000x magnification.

PLG microspheres manufactured using a double-emulsion method were characterised by SEM and laser diffraction particle sizing (Fig. 2). Particle sizing by laser diffraction revealed a particle size range of 20–100 μm diameter.

Assessment of dry microsphere morphology by SEM revealed a raisin-like spherical morphology and evidence of limited surface porosity. Microsphere suspension in aqueous and non-aqueous vehicles did not result in any apparent visual changes in surface characteristics, particle porosity, and morphology.

### Visualisation of vehicle and microsphere distributions by synchrotron x-ray micro computed tomography

**Fig. 3:**
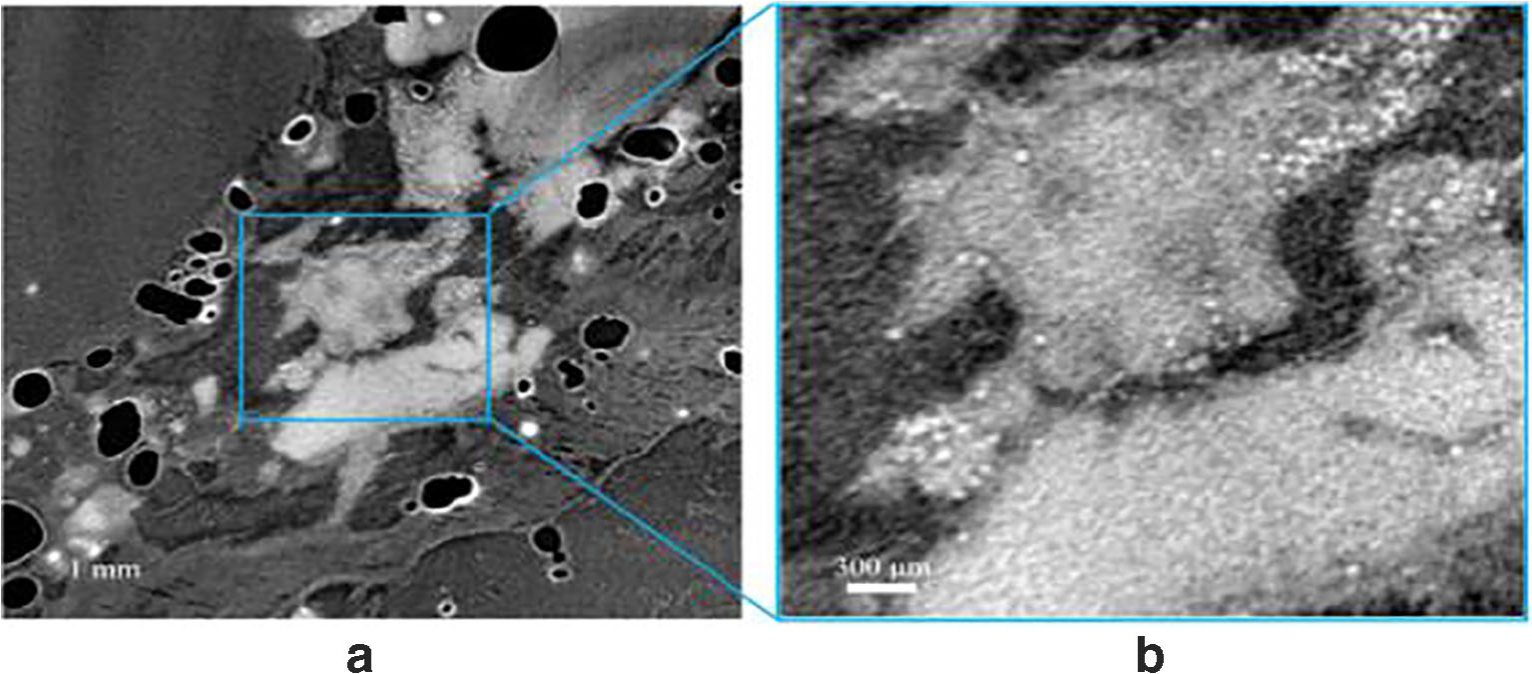
Reconstructed μCT data: representative cross-sectional slice from a standard reconstructed 3D dataset of microspheres and non-aqueous vehicle acquired using the module 1 setup.

Microspheres detected in the reconstructed μCT data were 3–4 pixels in diameter as a function of their size and image resolution, and exhibited varying visibility depending on the acquisition noise levels. Those microspheres deposited in a vehicle spiked with contrast agent were found to have brighter pixel values corresponding to higher attenuation levels, presumably as a consequence of contrast agent surface-adsorption (Fig. 3). Samples injected with aqueous vehicle in combination with the contrast agent gave a much more diffuse contrast than the nonaqueous vehicle meaning that tissue regions with low vehicle concentration were less visible.

In the absence of contrast agent, microspheres were detected albeit with reduced sensitivity.

Weakly X-ray absorbing and at a length scale marginally greater than the resolution of the system, the microspheres presented some challenges in automating image analysis. Material segmentation was not possible with intensity thresholding alone. However, it was possible to exploit the additional higher spatial frequencies within the unfiltered images due to the component of edge enhancement present, and consistent particle morphology, with a texture based custom thresholding method. When combined with intensity thresholding it was possible to significantly both increase the true positives and reduce the false positives of detected microspheres in the subcutis. Embedding the tissue samples in agarose made automatic segmentation challenging due to lack of density difference between the agarose and porcine tissue. Follow-up experiments, where samples were not embedded in agarose, significantly improved this process with no detrimental impact on data quality.

Visual assessment of microsphere deposition within various tissue samples revealed diffuse distribution of microspheres throughout tissue sections, with regions of concentrated microsphere depots observed near the needle track (i.e. the injection site).

During optimisation of tomographic image acquisition, the utility of Module 1 and the LFV module for the in-situ imaging of microspheres and vehicle was explored. The application of LFV module was considered since larger tissue sections can be imaged with the caveat of a lower resolution image being obtained. Overall, images generated from Module 1 were found to offer superior contrast and resolution with the capability to probe microsphere localisation when compared to the LFV setup.

### Three-dimensional reconstruction of tomographic images

**Fig. 4:**
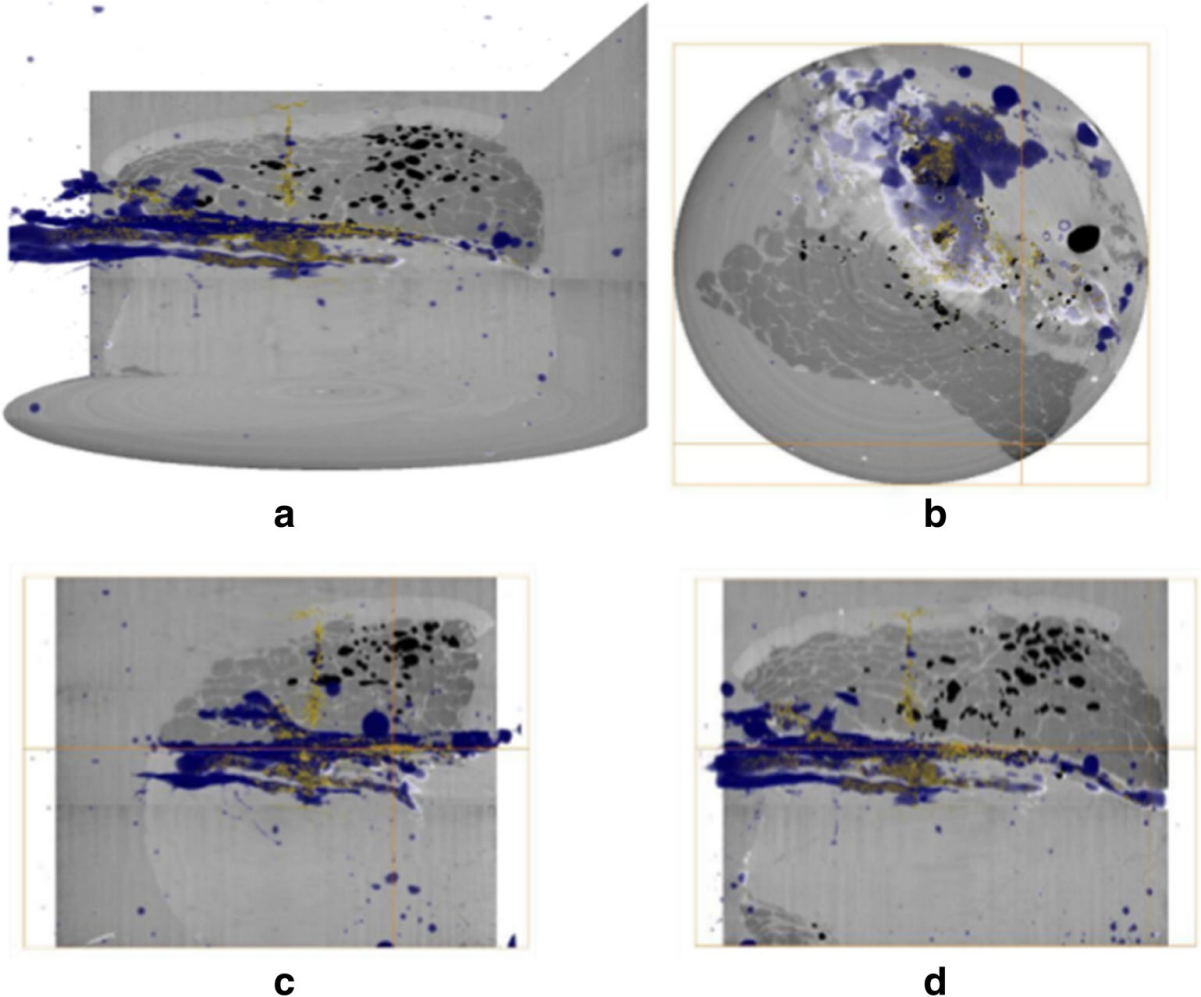
Three-dimensional visualisation of microsphere and non-aqueous vehicle deposition within porcine abdominal tissue acquired using the module 1 setup. Volume renderings of the microspheres (yellow) and contrast agent (blue) are overlaid onto orthogonal reconstructions of injected tissue samples. In (A) the perspective camera view is shown, and in each of (B), (C) and (D) the top, longitudinal and transverse views are presented orthographically. The vertical height shown is 18 mm, and the cross-sectional diameter 42 mm.

**Fig. 5:**
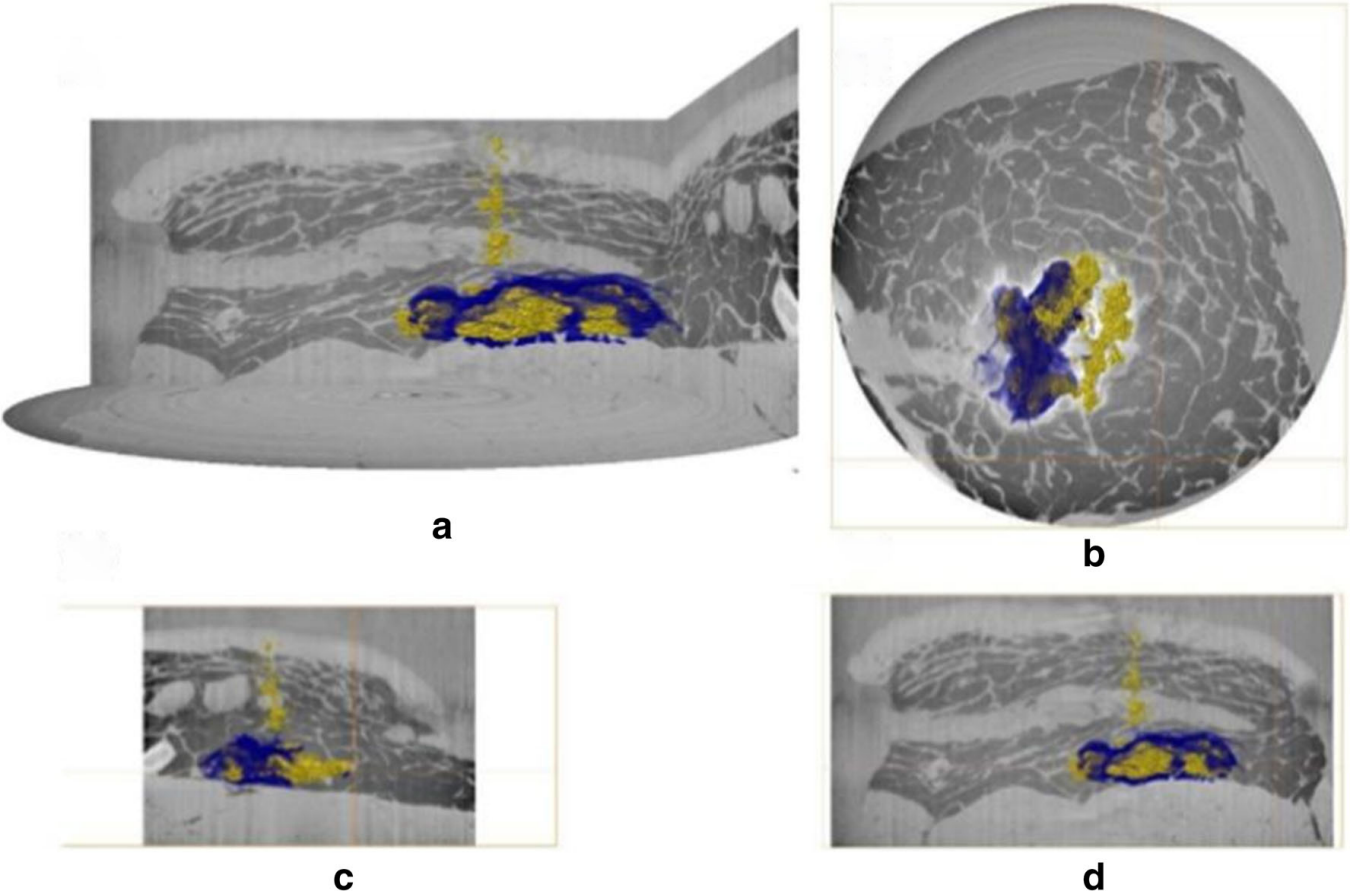
Three-dimensional visualisation of microsphere and aqueous vehicle deposition within porcine abdominal tissue acquired using the module 1 setup. Volume renderings of microspheres (yellow) and contrast agent (blue) are overlaid onto orthogonal reconstructions of injected tissue samples. In (A) the perspective camera view is shown, and in each of (B), (C) and (D) the top, longitudinal and transverse views are presented orthographically. The vertical height is 18 mm, and the cross-sectional diameter 42 mm.

Representative three-dimensional tomographic reconstructions, including volume renderings of microsphere and vehicle depot within porcine abdominal tissue are presented in Figs. 4 and 5.

Data presented above demonstrate the relative localisation of injected microsphere suspensions within excised porcine abdominal tissue. Microspheres and vehicle co-localised in some regions of the depot, and the presence of microspheres along the needle track close to the centre of injection was evident for both formulations. However, some degree of separation in tissue distribution was observed between the contrast-labelled vehicle and microspheres. The vehicle in both cases migrated to site distal to the centre of the injection, whereas, the microspheres were predominantly localized in proximity to the centre of the injection.

**Fig. 6:**
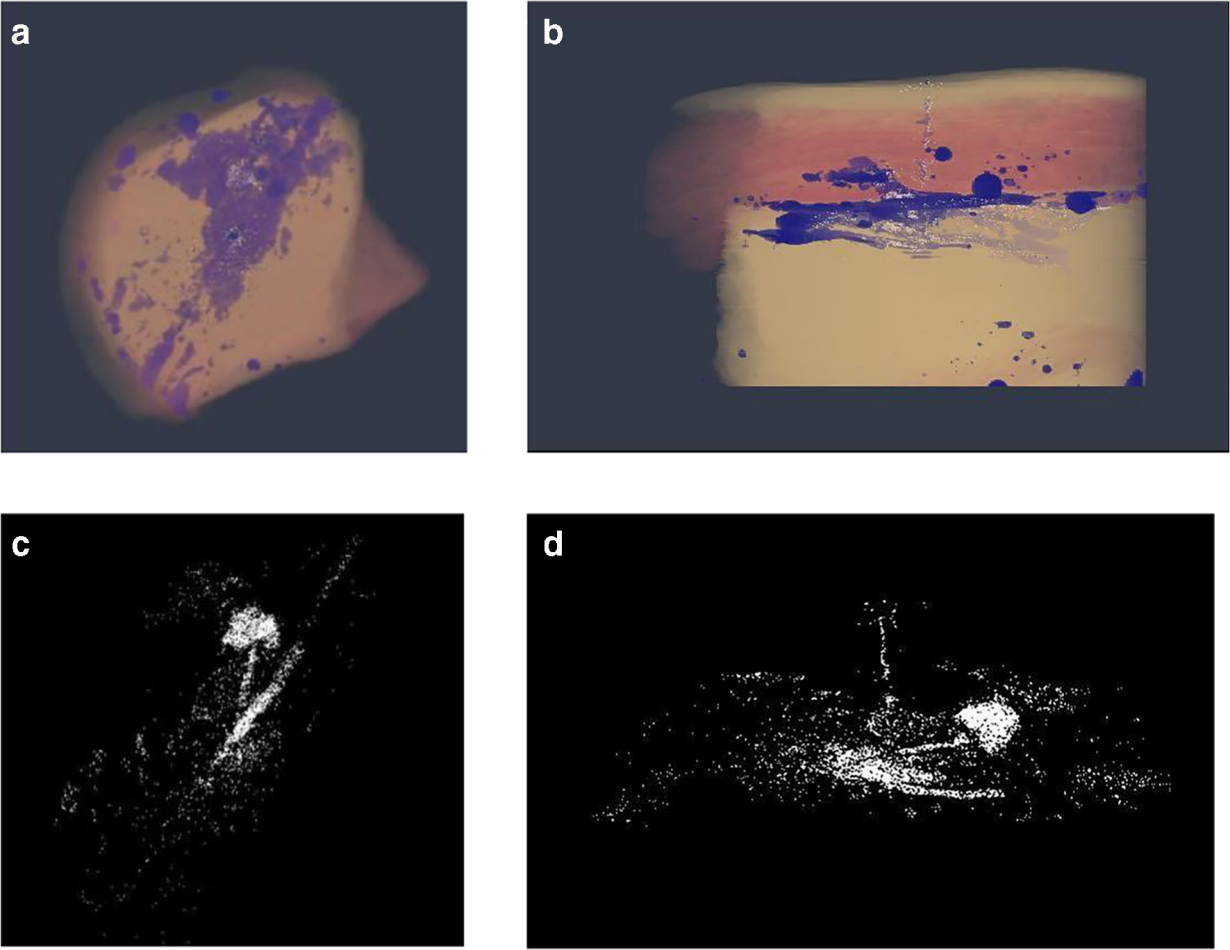
A three-dimensional snapshot of volume segmentation depicting aerial and transverse views respectively of the vehicle (A and B) and PLG microsphere distribution (C and D), within a representative tissue section.

A three-dimensional volume segmentation and rendering was also created for one representative sample, which is utilised in the graphical abstract and accompanying movie file. In Fig. 6, some stills are included which show the important features of the microsphere distribution within the porcine tissue.

Following image segmentation and removal of tissue and vehicle contribution (Fig. 6C-D), reconstructed microsphere depots show evidence of microsphere deposition along the needle tracks, with the bulk of microspheres located close to the centre of injection in a few clusters (depots). Some individual microspheres were located at distal sites within the excised tissue, consistent with the spread of injected (Fig. 6A-B).

**Fig. 7:**
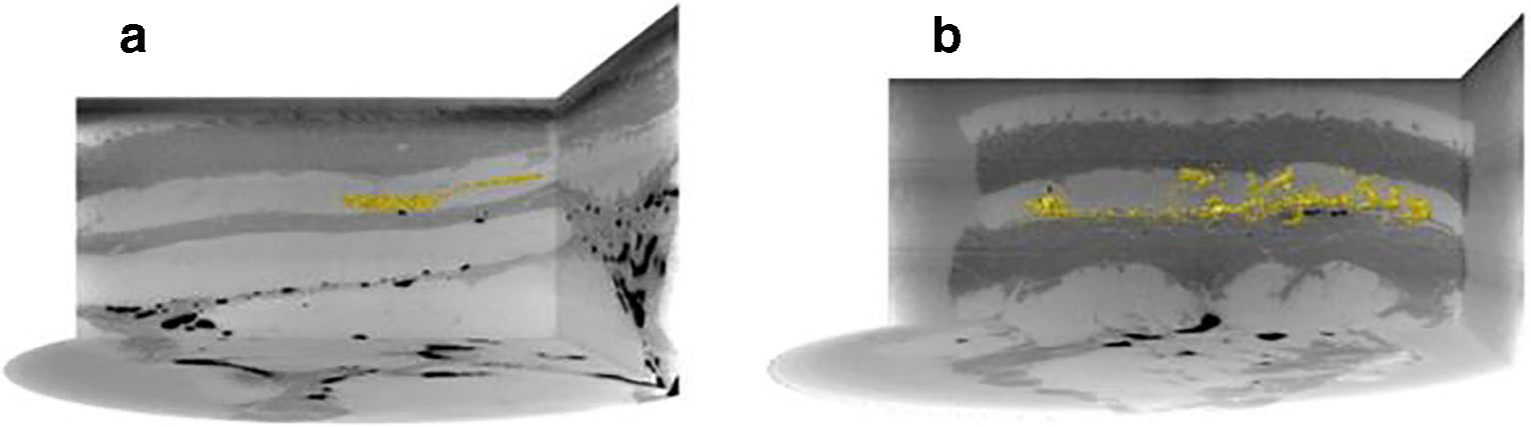
Volume renderings of PLG microspheres in the absence of contrast agent (yellow) are overlaid onto orthogonal reconstructed CT sections of the tissue sample, for (A) aqueous and (B) non-aqueous vehicles for images acquired using the LFV setup. The cross-sectional diameter represented here is 85 mm.

Volume representations of aqueous and non-aqueous vehicle depositions demonstrate microsphere identification (in yellow) in the absence of contrast agent (Fig. 7A and B). These were two of the larger porcine tissue samples imaged with the LFV camera module. Therefore, this technique could be applied in the absence of contrast agent, in scenarios where the density difference between microspheres and vehicle was sufficient, circumventing any associated pitfalls, for example, the impact of labelling on formulation rheology.

Volume renderings of PLG microspheres in the absence of contrast agent (Fig. 7) were created to evaluate the feasibility for microsphere depot detection in *ex vivo* porcine tissue. The benefit of this representation is to examine the relative anatomical localization of drug-loaded microspheres within tissue through simultaneous visualization of tissue layers and deposited microspheres without the need for a contrast agent.

### Visualisation of vehicle and microspheres by macrotomy and optical microscopy

Macroscopic assessment (i.e. photographic) of vehicle depot geometry was assessed in tissue sections injected with microsphere suspensions spiked with colorimetric dye (Patent Blue V and Solvent Green 3 for the aqueous and non-aqueous vehicles, respectively). To further visualise the three-dimensional spread of the vehicle within the subcutis, tissue background was removed, and depot reconstruction performed. Accurate two- and three-dimensional visualisation and quantification of vehicle spread for both vehicle types was achieved using this method.

**Fig. 8:**
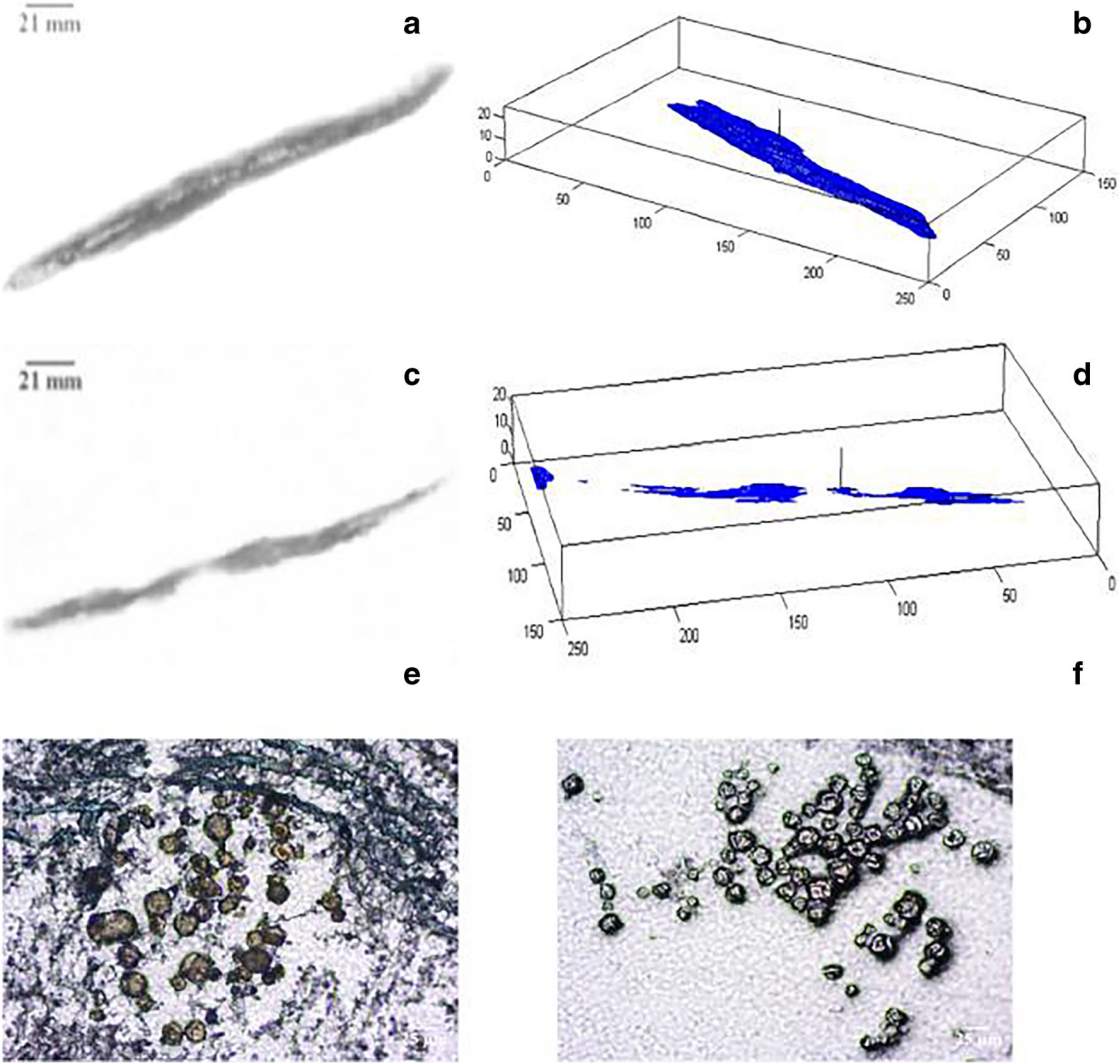
Representative two-dimensional sections (A and C), and reconstructed three-dimensional projections (B and D) of aqueous (A and B) and non-aqueous (C and D) vehicle depots. Typical brightfield micrographs of microsphere distribution within tissue sections for non-aqueous (E) and aqueous microsphere (F) suspensions (scale bar: 25 μm).

Microspheres were not detectable in the macroscopic (photographic) images acquired during macrotomy sectioning. Instead, every sixth tissue section was removed using adhesive tape and microspheres were visualised using optical microscopy. Microscopic assessment of PLG microsphere deposition revealed that microspheres were localised in pockets within tissue sections, and in close proximity to each other as evidenced in the presented micrographs Fig. 8E-F. Microsphere deposition was found to not always be co-localized with the vehicle depot in some cases. There was some evidence of tissue bursting around micrograph images, consistent with the presence of particles within the formulation.

## Discussion and conclusions

Controlled release microsphere suspension formulations are an attractive treatment paradigm in the routine management of a range of chronic conditions and offer the scope for improved patient adherence [23].

Probing formulation fate following *s.c.* injection offers the potential to understand the interplay between patient inter-individual variability in physiology, and formulation physicochemical attributes that can drive the design and development of novel formulations and devices leading to consistent successful therapeutic outcomes [24, 25].

Recent advances in high resolution imaging and tools for the characterisation of formulation material attributes have expanded the toolbox available to study formulation-related parameters and aid evaluation of their impact on biodistribution in the *s.c.* tissue.

The scope of the present study was to demonstrate the concept of synchrotron X-ray microcomputed tomography (μCT) in simultaneous two- and three-dimensional visualisation of suspension formulation component distribution in *ex vivo* porcine subcutis (*s.c.*). The formulations studied comprised PLG microspheres (20–100 μm diameter) suspended in an aqueous or a non-aqueous vehicle, in the presence and absence of a contrast agent.

We report the evaluation of PLG microsphere suspension depot fate post-injection using previously reported cryomacrotomy and synchrotron μCT. Macrotomy has previously been utilized to study insulin depot formation in subcutaneous tissue, demonstrating mostly non-spherical depot geometry. Injected insulin moves parallel to the skin surface across a path of least resistance in between adipose cells with depot geometry being dependent on tissue structure. At larger injection volumes (exceeding 0.1 mL), perpendicular movement relative to the skin surface occurs [15].

Macrotomy was implemented in this work to enable visualization of PLG microsphere and vehicle fate following injection (Fig. 8). However, the low resolution of dyed vehicle images posed a barrier to capturing the PLG microsphere depot using the same setup. Instead, sections collected during macrotomy were subjected to optical microscopy to evaluate relative microsphere and vehicle localization on a microscopic level. Findings from microscopic visualization and reconstruction of dyed vehicle depot, revealed similar observations to previous work in which both lateral movement of the vehicle across the tissue section was noted, with some evidence of backflow against the needle track (injection site) [15, 26]. In all samples there was evidence of significant microsphere retention in proximity to the centre of the injection site, and dyed vehicle migration in regions beyond microsphere localization. A key challenge with macrotomy was the labour-intensive process for collection of tissue sections for microscopic analysis of microsphere distribution. Subsequently, we performed high-resolution synchrotron μCT to enable the simultaneous visualization of vehicle and microsphere depot.

Readout from synchrotron μCT indicate that this approach can be used to visualize both microsphere and vehicle fate within *ex vivo *tissue sections in contrast to the macrotomy setup where two separate imaging approaches were utilized (Figs. 4 and 5). With the macrotomy approach we were unable to reconstruct the microsphere depot, significantly limiting assessment of post-injection formulation fate (Fig. 8). Another key limitation associated with this approach was potential freezing effects on tissue structure potentially impacting microsphere and vehicle localization, and sectioning or removal of microspheres during macrotomy. Findings from reconstructed μCT images showed co-localization of microsphere (yellow) and vehicle (blue) along the needle track and in proximity to the centre of the injection site where the bulk of microspheres accumulated. Taken together with observations from cryomacrotomy, these findings confirm that tissue structure restricts microsphere movement by acting as a sieve, such that most injected microspheres are retained close to the centre of the injection site (Fig. 6C-D), and the injected vehicle flows along the path of least resistance (Figs. 4, 5, 6). These observations are in close agreement with previous observations of suspension formulation fate described by Rasmussen et al [27, 28]. The vehicle was observed to migrate to distal sites in the *ex vivo* tissue section consistent with previous findings on suspension injection fate where only a small number of particles are deposited at sites distal to the centre of injection (Fig. 6C-D).Using μCT we also visualized the relative anatomical deposition of microspheres and vehicle within excised tissue.

Lab-based CT has previously been utilised to visualise and quantify spatial distribution of soluble insulin formulations [26, 27]. We selected synchrotron μCT for these experiments because with an effective voxel size of 50 μm and resolution of 120 μm, resolution of microspheres in the 20–100 μm size range could not be achieved with conventional lab-based CT. Furthermore, the image acquisition time for lab-based CT is significantly longer for the tissue section sizes investigated in this study (45 min *versus* 50 or 150 s). The resolution of the technique reported here enabled the detection of individual deposited PLG microspheres (20–100 μm diameter) within *ex vivo* porcine tissue, and visualization of subcutaneously- injected microsphere suspension fate.

A component of the PLG microsphere visibility may be attributed to the additional phase contrast present (edge enhancement- where each material interface is highlighted by a local intensity minimum directly adjacent to the maximum). This is one of the several advantages of using a synchrotron X-Ray source for the CT (other advantages include improved contrast from the smaller energy bandwidth, shorter acquisition times possible due to higher photon flux, etc).

Acquisition of freshly euthanised/excised tissue would be recommended before drawing conclusions on the impact of formulation variables (volume, injection force, vehicle viscosity, lipophilicity etc.) on suspension formulation depot geometry, since changes in mechanical properties of the tissue as a result of temperature, hydration status and exposure to air likely reduce its *in vivo* relevance. All *ex vivo* tissues sourced for these experiments were acquired from an abattoir within hours of euthanasia.

As with the tomography approach used by Thomsen * et al.*, the flexibility to segment various components enabled study of elements of interest (e.g. vehicle, microspheres, and tissue layers) [26].

In contrast to benchtop CT, the CT method and image analysis approach described here requires optional addition of contrast agent to the vehicle, without the need for resource intensive remanufacture of polymeric microspheres incorporating contrast agent or label, which can often be prohibitive in such imaging studies. The uniform shape of the microspheres, in combination with the high resolution of synchrotron μCT, enabled visualization of individual microsphere particles within the *ex vivo* tissue section in the presence and absence of contrast agent, circumventing any caveats associated with contrast agent effects on vehicle rheology and properties. The three-dimensional microsphere depot was reconstructed in the presence or absence of background tissue elements (see supplementary video) to enable assessment of relative anatomical localisation of formulation components.

The true power of synchrotron μCT in combination with the image analysis approach developed here is visualisation of the *in situ* microsphere depot through edge enhancement, without the need for contrast agent. This is one of the several advantages associated with using a synchrotron X-Ray source for the CT. Other advantages of this approach included rapid image acquisition times due to higher photon flux, and improved contrast from the smaller energy bandwidth.

Since tissue excision is currently a pre-requisite for high resolution μCT imaging of formulation deposition, the technique is limited to *ex vivo* tissue samples. A detailed account of depot geometry impact on *in vivo* modelling of drug absorption is not within the scope of this manuscript. While findings from the present manuscript cannot be directly linked to the PK behaviour of injected PLG microspheres, the additional insights gained into injection site depot geometry could feasibly contribute to development of predictive *in vitro* or mechanistic in silico models of subcutaneously- injected formulations in combination with dissolution data. Findings from this work could also contribute to a more thorough understanding of the role of depot geometry on interspecies and interindividual differences in the rate and extent of absorption, and impact of formulation variants or variability in pharmacokinetics. To our knowledge there are no reports of PLG microsphere suspension formulation *in situ* depot geometry using synchrotron CT in the published literature.

Based on the successful outcome of this *proof of concept* study, *in vivo* studies performed with PK sampling followed by euthanasia and excision of the injection site in an appropriately-designed timepoint preclinical study may be used to link depot geometry findings directly to *in vivo* PK. Coupled with appropriate pharmacokinetic analyses of plasma concentrations, and histological evaluation of immune cell infiltration, this approach could foreseeably be employed to mechanistically investigate the link between depot geometry and the rate/extent of absorption during formulation and device development.

This technique may also be applied to other routes of administration including intramuscular injection, and alternative formulation types (*in situ* forming depots or crystalline/amorphous microsuspensions).

With future work, and optimisation of experimental parameters and image processing, microsphere detection could be improved to enable greater automation and accuracy. Machine learning tools for segmentation could also potentially be applied to aid this process. Once accurate segmentation and labelling is complete, tomographic analysis allows for almost unlimited statistical measurement of volume-based distributions.

### Acknowledgements and Disclosures

The authors would like to thank Ashton Gwinnell, David Leonard, Karolina Dziemedowicz and Kathryn Pickup for their assistance in method development and sample preparation, Gavin Reynolds for his work on digital image reconstruction and Per-Ola Norrby for his assistance in preparation of the manuscript. At Diamond Light Source we are grateful to Robert Atwood and Nghia Vo for their support during synchrotron beamtime and afterwards with the tomographic reconstructions. This work was funded by AstraZeneca Pharmaceuticals. All experiments were performed using porcine tissue sourced from ethically responsible abattoirs. This work has not been published or under consideration for publication elsewhere. All authors have reviewed and approved the content of this manuscript.

## Electronic supplementary material

ESM 1 (DOCX 671 kb)

## References

[1] DeYoung MB, MacConell L, Sarin V, Trautmann M, Herbert P (2011). Encapsulation of exenatide in poly-(D,L-lactide-co-glycolide) microspheres produced an investigational longacting once-weekly formulation for type 2 diabetes. Diabetes Technol Ther.

[2] Gefvert O, Eriksson B, Persson P, Helldin L, Björner A, Mannaert E, Remmerie B, Eerdekens M, Nyberg S (2005). Pharmacokinetics and D2 receptor occupancy of long-acting injectable risperidone (Risperdal Consta™) in patients with schizophrenia. Int J Neuropsychopharmacol.

[3] C.f.s.a. treatment, Treatment Improvement Protocols, in: S.a.a.m.h.s. administration (Ed.) Incorporating Alcohol Pharmacotherapies into Medical PracticeRockville, MD, USA, 2009.

[4] Petersen H, Bizec J-C, Schuetz H, Delporte M-L. Pharmacokinetic and technical comparison of Sandostatin® LAR® and other formulations of long-acting octreotide. BMC Research Notes. 2011;4:344–4. 10.1186/1756-0500-4-344 PMC3212992 21906300

[5] Zuidema J, Kadir F, Titulaer HAC, Oussoren C (1994). Release and absorption rates of intramuscularly and subcutaneously injected pharmaceuticals (II). Int J Pharm.

[6] Fathallah AM, Balu-Iyer SV (2015). Anatomical, physiological and experimental factors affecting the bioavailability of sc administered large biotherapeutics. J Pharm Sci.

[7] Richter WF, Jacobsen B (2014). Subcutaneous absorption of biotherapeutics: knowns and unknowns. Drug metabolism and disposition: the biological fate of chemicals.

[8] Richter WF, Bhansali SG, Morris ME (2012). Mechanistic determinants of biotherapeutics absorption following SC administration. AAPS J.

[9] Schwendeman SP, Shah RB, Bailey BA, Schwendeman AS (2014). Injectable controlled release depots for large molecules. Journal of controlled release: official journal of the Controlled Release Society.

[10] Zhao M, Rodríguez-Villagra E, Kowalczuk L, Le Normand M, Berdugo M, LevyBoukris R, El Zaoui I, Kaufmann B, Gurny R, Bravo-Osuna I, Molina-Martínez IT, Herrero-Vanrell R, Behar-Cohen F (2017). Tolerance of high and low amounts of PLGA microspheres loaded with mineralocorticoid receptor antagonist in retinal target site. J Control Release.

[11] Liu R, Zhang M, Jin C (2017). In vivo and in situ imaging of controlled-release dissolving silk microneedles into the skin by optical coherence tomography. J Biophotonics.

[12] Kim D-H, Li W, Chen J, Zhang Z, Green RM, Huang S, Larson AC (2016). Multimodal imaging of Nanocomposite microspheres for Transcatheter intra-arterial drug delivery to liver tumors. Sci Rep.

[13] Li ZY, Qin XY, Guo LY, Wang H, Liu XX, Zheng ZZ, Guan HT, Song L, Zou YH, Fan TY (2017). Poly(acrylic acid) microspheres loaded with superparamagnetic iron oxide nanoparticles for transcatheter arterial embolization and MRI detectability: in vitro and in vivo evaluation. Int J Pharm.

[14] Zhou H, Hernandez C, Goss M, Gawlik A, Exner AA (2015). Biomedical imaging in implantable drug delivery systems. Curr Drug Targets.

[15] Jockel JP, Roebrock P, Shergold OA (2013). Insulin depot formation in subcutaneous tissue. J Diabetes Sci Technol.

[16] Swindle MM, Makin A, Herron AJ, Clubb FJ, Frazier KS (2012). Swine as models in biomedical research and toxicology testing. Veterinary Pathology Online.

[17] M. Drakopoulos, T. Connolley, C. Reinhard, R. Atwood, O. Magdysyuk, N. Vo, M. Hart, L. Connor, B. Humphreys, G. Howell, S. Davies, T. Hill, G. Wilkin, U. Pedersen, A. Foster, N.De Maio, M. Basham, F. Yuan, K. Wanelik, I12: the joint engineering, environment and processing (JEEP) beamline at diamond light source, J Synchrotron Radiat, 22 (2015) 828–838. 10.1107/S1600577515003513 PMC4416690 25931103

[18] R. Fitzgerald, Phase-sensitive X-ray imaging, physics today, 53 (2000).

[19] R.C. Atwood, A.J. Bodey, S.W. Price, M. Basham, M. Drakopoulos, A high-throughput system for high-quality tomographic reconstruction of large datasets at Diamond Light Source, Philosophical transactions. Series A, Mathematical, physical, and engineering sciences, 373 (2015). 10.1098/rsta.2014.0398 PMC4424489 25939626

[20] Vo NT, Atwood RC, Drakopoulos M (2015). Radial lens distortion correction with sub-pixel accuracy for X-ray micro-tomography. Opt Express.

[21] Paganin D, Mayo SC, Gureyev TE, Miller PR, Wilkins SW (2002). Simultaneous phase and amplitude extraction from a single defocused image of a homogeneous object. J Microsc.

[22] Leuenberger Jockel JP, Roebrock P, Shergold OA (2013). Insulin depot formation in subcutaneous tissue. J Diabetes Sci Technol.

[23] Wysham CH, Rosenstock J, Vetter ML, Dong F, Öhman P, Iqbal N (2018). Efficacy and tolerability of the new autoinjected suspension of exenatide once weekly versus exenatide twice daily in patients with type 2 diabetes. Diabetes Obes Metab.

[24] Mathaes R, Koulov A, Joerg S, Mahler H-C (2016). Subcutaneous injection volume of biopharmaceuticals—pushing the boundaries. J Pharm Sci.

[25] Horejsi R, Möller R, Pieber TR, Wallner S, Sudi K, Reibnegger G, Tafeit E (2002). Differences of subcutaneous adipose tissue topography between Type-2 diabetic men and healthy controls. Exp Biol Med.

[26] Thomsen M, Rasmussen CH, Refsgaard HHF, Pedersen K-M, Kirk RK, Poulsen M, Feidenhans'l R (2015). Spatial distribution of soluble insulin in pig subcutaneous tissue: effect of needle length, injection speed and injected volume. Eur J Pharm Sci.

[27] Rasmussen CH, Roge RM, Ma Z, Thomsen M, Thorisdottir RL, Chen JW, Mosekilde E, Colding-Jorgensen M (2014). Insulin aspart pharmacokinetics: an assessment of its variability and underlying mechanisms. Eur J Pharm Sci.

[28] C.H. Rasmussen, T. Soeborg, E. Mosekilde & M. Colding-Jorgensen. Absorption Kinetics of Insulin Mixtures after Subcutaneous Administration. In: MOSEKILDE, E., SOSNOVTSEVA, O. & ROSTAMI-HODJEGAN, A. (eds.) Biosimulation in Biomedical Research, Health Care and Drug Development. (2012) Vienna: Springer Vienna.

