# Supplementary material for: Novel application of synchrotron x-ray computed tomography for ex-vivo imaging of subcutaneously injected polymeric microsphere suspension formulations
Source: Pharm Res. 2020 May 14;37(6):97. doi: 10.1007/s11095-020-02825-9 (PMC7225200; doi:10.1007/s11095-020-02825-9)
Supplement: Supplementary file 1 — (DOCX 671 kb) [file 11095_2020_2825_MOESM1_ESM.docx]

# SUPPLEMENTARY INFORMATION µCT Camera module information

For image acquisition with detector module 1 and the smaller porcine tissue samples, experiments were conducted in experimental hutch 1 (EH1) at the I12 beamline, located around 50 m from the X-ray source. The LFV detector module is located in EH2, roughly 100 m from the source (and inline with EH1). Here the available beam size has approximately twice both the width and height (97 mm (H) x 25 mm (V)) due to the divergence of the X-ray beam. The larger field of view is better suited for the larger porcine tissue samples. As they are correspondingly thicker along the X-ray path and significantly more attenuating, longer exposure times were required. To increase the dynamic range of the image acquisition, a ‘bowtie filter’ was also utilised. This is a beam shaping filter designed to homogenise the beam profile reaching the detector. In our case this was composed of a PMMA cylinder (of diameter fit roughly to the tissue size) within a PMMA box, whereby the volume exterior to the cylinder was filled with a CaCO3 powder. Its geometry compensates for thick cylindrical and strongly attenuating samples which would otherwise cause detector saturation at the sample periphery, permitting longer exposure times and therefore improved signal to noise levels.

The two modules require different pre-processing steps. The lens system for Module 1 exhibits a characteristic barrel distortion which is corrected for following distortion measurement with a calibration grid [19]. The LFV module requires stitching of two separately-acquired image sets (with merging of the overlap region).

# µCT image processing

A schematic overview of the image processing and reconstruction steps for both modules is shown in Fig. S1. For each module, two separate tomographic reconstructions are performed (standard and phase retrieval-based), and both sets of reconstructed data are then used as input for the microsphere assessment and marking workflow (Fig. S2). An example of the resulting mapping process is shown in Fig. S3. In some cases, the location of a microsphere is indicated only by a central (brightest) pixel, and in this sense, it is not a true segmentation of the microsphere material.

Flat field

correction

Lens distortion correction (

**module 1**

/merge 2

)

camera views (

**LFV module**

)

Phase retrieval

Paganin filter)

(

Ring removal

Phase

-

based

tomographic

reconstruction

combine stacks

for visualisation

Ring removal

Absorption

-

based

tomographic

reconstruction

Combine stacks

for visualisation

**Fig. S1.** Processing and reconstruction schematic for the two camera modules for µCT

visual inspection of

combined stack of phase

retrieved slices to

determine range of slices

containing microspheres

sample slices at regular

intervals, marking ROI

within those slices

manually

interpolate ROI

dimensions over all slices

containing microspheres.

Then for each slice ROI,

compute:

gradient + heavy

gaussian filter + open

to create mask

marking clusters of

microspheres

intensity thresholding

(

thresholds manually

determined for each

sample)

combine conditions above to generate spot maps marking microspheres.

Visually confirm positions are predominantly marking microspheres

and not noise. If not, repeat thresholding above with varied levels.

bandpass filter,

correponding to typical

bead size, for texture

thresholding (manually

chosen for each sample)

intensity thresholding of

phase retrieved slices

manually deteremined

)

(

**Fig. S2.** Schematic of the process required to assess and mark microsphere deposition within tissue sample µCT data. All software was custom-written in IDL.


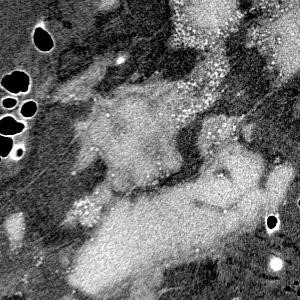

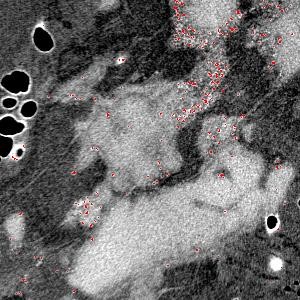

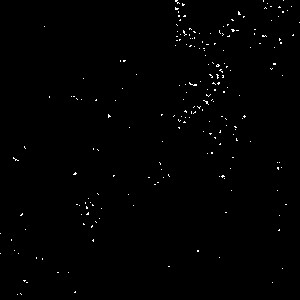


**Fig. S3.** Microsphere detection and marking within a 5.5 mm × 5.5 mm region of interest within a tomographic slice of the reconstructed µCT data. With non-aqueous vehicle deposition within porcine abdominal tissue acquired using the module 1 setup. The microspheres, detected via the process outlined in Fig. S2, are marked in red in (b), with the resulting isolated map in (c).

Representative bright-field micrographs of microsphere distribution within macrotomy tissue sections are presented in **Fig. S4** and **Fig. S5**.


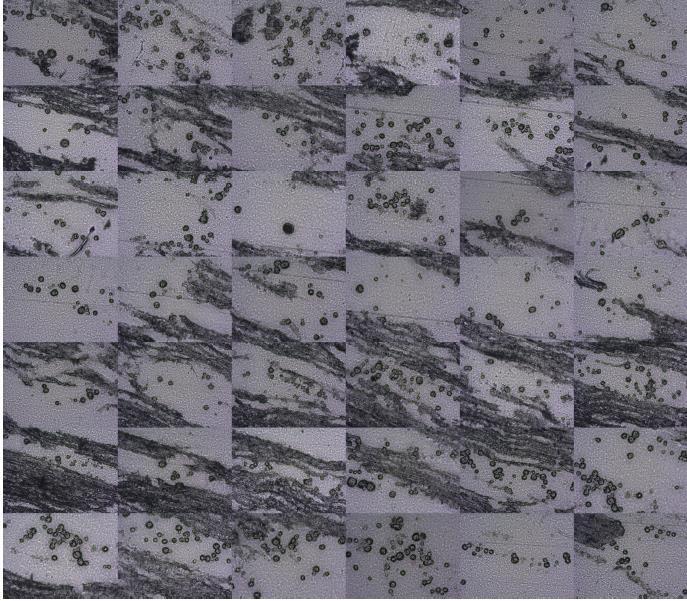


**Fig. S4.** A montage of representative micrographs acquired across the length of a tissue section for injected with non-aqueous microsphere suspension. The stitched images are presented in no particular order along the length of the depot.


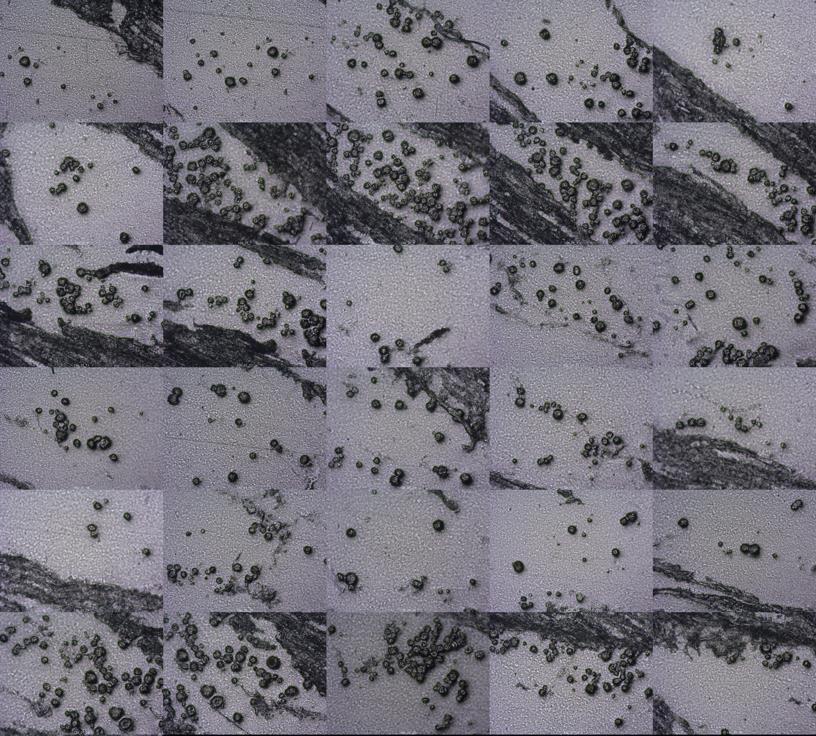


**Fig. S5.** A montage of representative micrographs acquired across the length of a tissue section injected with aqueous microsphere suspension. The stitched images are presented in no particular order along the length of the depot.
